# Dynamic Effects of Laser Action on Quasi-Two-Dimensional Dusty Plasma Systems of Charged Particles

**DOI:** 10.3390/molecules25153375

**Published:** 2020-07-25

**Authors:** Mikhail M. Vasiliev, Oleg F. Petrov, Anastasiya A. Alekseevskaya, Alexander S. Ivanov, Elena V. Vasilieva

**Affiliations:** 1 Joint Institute for High Temperatures, Russian Academy of Sciences, 115478 Moscow, Russia; ofpetrov@ihed.ras.ru (O.F.P.); nastya-mipt@yandex.ru (A.A.A.); asi.kiae@gmail.com (A.S.I.); elen_vasilieva@mail.ru (E.V.V.); 2 Moscow Institute of Physics and Technology, 141700 Dolgoprudny, Russia; 3 NRC “Kurchatov Institute”, Kurchatov Sq., 1, 115478 Moscow, Russia

**Keywords:** colloidal plasma, two-dimensional structure, dusty plasma flow, photophoretic force

## Abstract

We present the results of an experimental study of the behavior of a colloidal plasma system formed by copper-coated and uncoated polymer particles under the action of laser irradiation. A comparative study of particle velocity distribution profiles depending on the power of the pushing laser was conducted. In the case of uncoated melamine-formaldehyde (MF) particles, we observed the well-known action of light pressure, causing shear stress in the colloidal plasma structure and leading to the occurrence of a laminar flow within the affected area. For the copper-coated MF particles, we revealed some additional patterns of behavior for the dust particles, i.e., kinetic temperature growth due to laser radiation absorption by the copper coating, as well as the appearance of chaotic particle motion. We believe that this happens due to the existence of defects in the coating, causing asymmetric heating of the particles, which in turn leads to chaotic deviations of the photophoretic force pushing the particles in different directions.

## 1. Introduction

The phenomena of self-organization in nature are extremely diverse and occur in systems of different complexities and scales-from the physical systems studied in both the nanoworld and in astronomy, to the social and economic processes in human society. Self-organization is an inherent feature of open non-equilibrium systems, involving non-linear interactions of their components. As an example of such systems, one can mention colloidal plasma formed by micron-sized charged particles, confined in the plasma of an electric gas discharge. The intense scattering of laser radiation by particles makes it easy to observe and investigate systems formed by charged dust particles by tracking their coordinates and speeds in real time. For this reason, colloidal plasma is a convenient tool for studying various phenomena, such as phase transitions or flow formation.

An external influence such as laser radiation is widely applied in experiments to control the degree of order and spatial positions of the particles. Using this technique, laminar flows in a dusty plasma liquid can be generated [1]; the viscosity properties, such as the coefficients of shear viscosity and shear stress, can be determined [2,3]. In previous work [4,5], the Brownian motion of dust grains in plasma was investigated by video recording of the particle dynamics, so that the friction coefficient of dust particles in the buffer gas was measured. One can also use lasers to heat dust particles and increase their kinetic energy due to the photophoretic force; thus, phase transitions in colloidal plasma systems can be observed [6,7].

It was shown that the kinetic energy (and therefore kinetic temperature) of dust particles can be smoothly changed with a wide range of coupling parameters Γ* [8]. There are at least two ways to change the kinetic temperature. The first one is to change the pressure of the plasma-forming gas, *P_n_*. This directly affects the friction force *F_fr_*, acting on the dust particles [9]. In the free molecular mode at low dust particle speeds (*u_d_* << *v_Tn_),* this force is equal to:

(1) $$F_{fr}\sim \;r_{d}^{2}P_{n}\left(\frac{u_{d}}{v_{T_{n}}}\right),$$

where *r_d_* is the particle radius, *u_d_* is the average particle velocity, and *v_Tn_* is the average velocity of the atoms of the plasma-forming gas. In this case, the average velocity of the dust particles is inversely proportional to the pressure of the buffer gas. This method of changing the kinetic temperature was observed in [10], while studying the dependence of the colloidal plasma viscosity on the value of the coupling parameter.

Another way to influence the kinetic temperature of dust particles in plasma is to heat them with laser radiation and to increase their speed using photophoretic force. It should be noted that melamine-formaldehyde (MF) polymer particles, which are used in a number of our experiments, barely absorb laser radiation. Therefore, there is no increase in the kinetic temperature of the dust component during laser irradiation [11]. By contrast, particles with a, for instance, metal coating absorb laser radiation effectively, which in turn leads to their heating. 

In this study the flow formation and evolution in a monolayer crystal-like structure for different types of particles in RF discharge plasma (at pressure P = 5.3 Pa and RF discharge power W_rf_ = 12.5 W) due to effect of laser radiation is presented.

## 2. Data Analysis and Discussion

### 2.1. Materials

**Figure 1:**
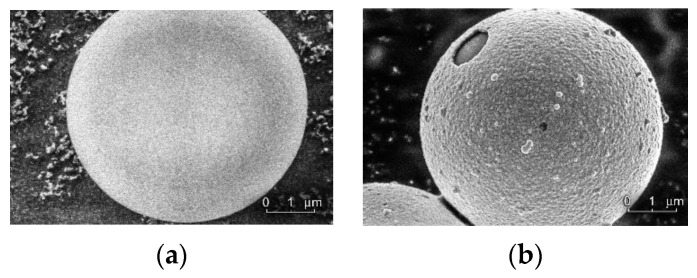
SEM photographs of a spherical monodisperse melamine-formaldehyde (MF) particle: (**a**) with a diameter of 9.95 μm, without coating; (**b**) with a 200-nm-thick copper coating.

In the experiments, we used two types of monodisperse melamine-formaldehyde (MF) spheres: one with a density of *ρ*_p_ = 1.61 g/cm^−3^ and a diameter of 9.95 μm as a dust component, without any coating; and another with a surface covered by 200 nm of copper (see Figure 1). To detect possible erosion of the particle surfaces in the plasma or as a result of exposure to laser radiation, the particles were observed before and after the experiment by means of scanning electron microscopy. No changes in the structure or continuity of the coating were found, as opposed to work [12] in which laser with power in three orders of magnitude higher than in our experiments damaged the surface of particles.

As noted above, MF particles practically do not absorb light at a wavelength of 532 nm. However, after covering the particles with a thin copper coating, their absorption capacity increases dramatically. If such particles are exposed by laser radiation, their surfaces will be heated up to ΔT ~ 10–100 K, as estimates show. Thus, laser radiation can lead to at least two observed effects. Firstly, according to [10], there will be a noticeable increase in the kinetic temperature of the particles under the action of a photophoretic force, and secondly the directional flow will be observed. The photophoretic force, according to [13], depends on the thermal conductivity of dust particles and is equal to:

(2) $$F_{ph}=\frac{\pi r_{d}^{3}P_{n}}{6æT_{n}}I,$$

where *æ* is particle thermal conductivity and *I* is the luminous flux density.

The thermal conductivity of melamine-formaldehyde *æ*_mf_ ≈ 0.2 W/(m·K) is rather small, compared to the thermal conductivity of the copper coating *æ*_Cu_ ≈ 400 W/(m·K), that is three orders of magnitude larger. Consequently, the temperature distribution on the surfaces of the particles, which is a cause of the photophoretic force, will be determined mainly by the thermal conductivity of copper. An elementary estimate shows that the characteristic time for temperature equalization on the surfaces of the particles is ~10^−6^ s. The heating of the inner region of the particle occurs after ~10^−3^ s. In addition, the temperature distribution can be affected by the heterogeneity of the copper coating, which takes place according to Figure 1. Therefore, the direction of the photophoretic effect for different particles may not quite coincide with the direction of the laser beam.

### 2.2. Experimental Data

We observed flow formation for a structure formed by uncoated particles, while varying the power of the pushing laser (see Section 3) beam in the range of 0–250 mW. The power of the illuminating laser (see Section 3) was about ~100 mW, so that particles were clearly detectable by video camera. Before starting the laser exposure, we estimated for each set of experiments the magnitude of the effective coupling parameter for the unperturbed monolayer structure formed by uncoated MF particles. This estimation was based on the analysis of the first maximum of the pair correlation function of the dust structure [14]. In our experiments, the effective coupling parameter was Γ* ~ 500. We should mention that an increase of the illuminating laser power up to 100 mW did not influence the particle motion or phase state of structure (and the value of Γ*), since uncoated particles barely absorbed laser radiation.

**Figure 2:**
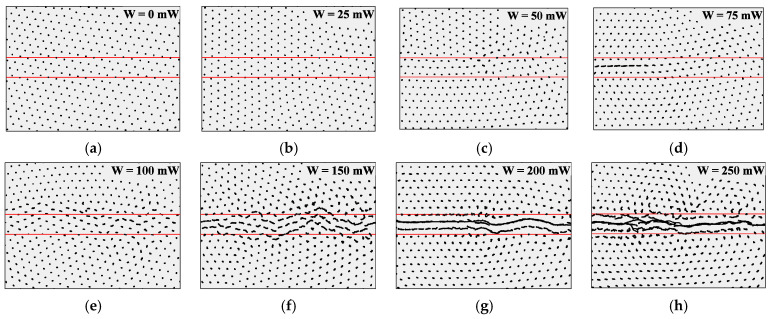
Particle trajectories in the dust structure (Γ* = 500), consisting of uncoated MF particles in argon at a pressure of P = 5.3 Pa and a discharge power of W_rf_ = 12.5 W during t = 0.5 s under the action of a pushing laser beam with a width of ~4 mm and laser powers of: (**a**) W = 0 mW; (**b**) W = 25 mW; (**c**) W = 50 mW; (**d**) W = 75 mW; (**e**) W = 100 mW; (**f**) W = 150 mW; (**g**) W = 200 mW; (**h**) W = 250 mW.

**Figure 3:**
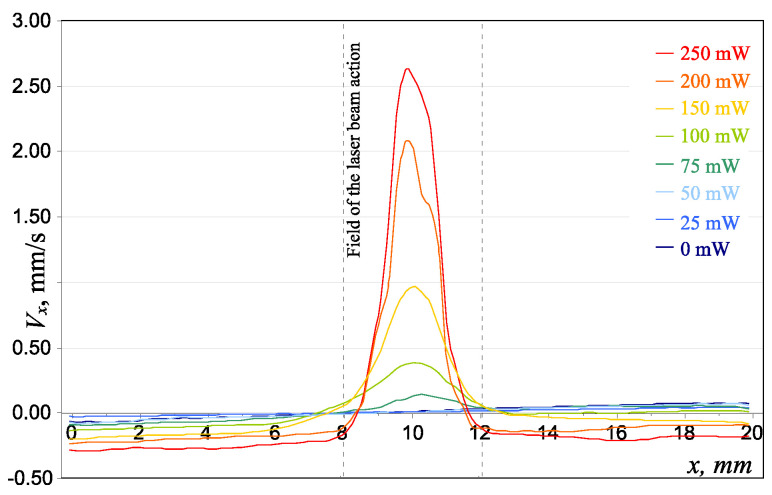
The velocity distribution profile for MF particles in the monolayer dust structure under the action of the pushing laser with power values of W = 0, 25, 50, 75, 100, 150, 200, and 250 mW.

Figure 2 presents the trajectories of dust particles in the structure over a period of 0.5 s for various pushing laser power values. Solid red lines define boundaries of the area affected by the pushing laser beam. Figure 3 shows the velocity distribution profile for uncoated MF particles in the monolayer dust structure under the action of the pushing laser, with a power W varying from 0 to 250 mW.

From the analysis of the trajectories of the particle motions (see Figure 2), as well as of the distribution of their velocities in the structure (see Figure 3), we can consider the threshold nature of the flow, which is consistent with the experimental results obtained in [6,7]. Under the influence of a pushing laser with a power close to the critical value, we observed a deformation of the structure, however the flow still did not occur. With a further increase in the power of the laser radiation, the formation of a directional motion of dust particles in the region of the laser beam exposure was observed.

In the experiment with the copper-coated MF particles, the illuminating laser power was chosen to be as weak as possible, so as not to heat the particles via radiation absorption, but still strong enough for illumination in order to detect particles via video camera (~36 mW). With similar discharge parameters of P = 5.3 Pa and W_rf_ = 12.5 W, the effective coupling parameter for the unperturbed dust structure, which was reconstructed from the first peaks of the pair correlation function, was Γ* ~ 180. This value of Γ* is considered to be an indicator of a crystalline type of structure.

**Figure 4:**
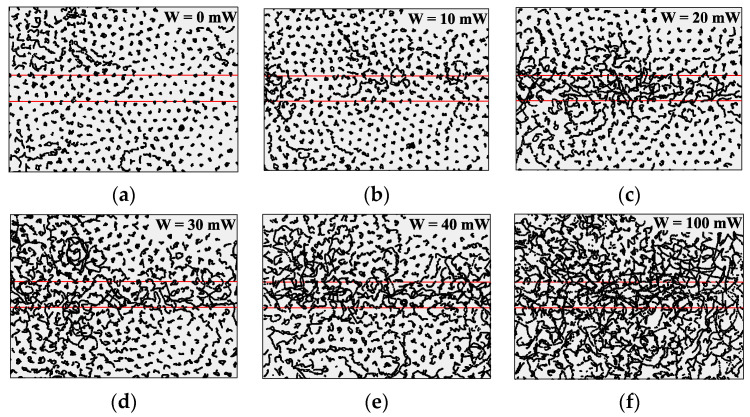
Particle trajectories in the monolayer dust structure (Γ* ~ 180), which consisted of copper-coated MF particles in argon at a pressure of P = 5.3 Pa and a discharge power of W_rf_ = 12.5 W during t = 0.5 s under the action of a pushing laser beam with a width of ~4 mm and laser power values of W: (**a**) W = 0 mW; (**b**) W = 10 mW; (**c**) W = 20 mW; (**d**) W = 30 mW; (**e**) W = 40 mW; (**f**) W = 100 mW.

Figure 4 presents the trajectories of dust particles in a monolayer dust structure formed by copper-coated MF particles during t = 0.5 s under the action of a pushing laser beam with power values W varying from 0 to 100 mW. We should note that the side view camera did not detect any amplitude gain of vertical particle motion.

### 2.3. Data Analysis 

In order to study the difference in the laser’s effects on the dust structure for particles with and without a copper coating, let us estimate the magnitudes of the force of light pressure, photophoretic force, and threshold pressure needed for a flow to occur in the structure under consideration.

Light pressure *P_γ_* is determined by radiation power *W*, as indicated in [15]

(3) $$P_{\gamma }=\frac{W\left(1+\theta \right)}{S_{\gamma }c}\;,$$

where *θ* is a coefficient varying from 0 to 1, depending on the nature of the reflection of light from the surface of the particle; *S_γ_* is a sectional area of the light beam; and *c* is the light’s velocity. Corresponding to this pressure, the force acting on the particle is equal to *P_γ_S_d_*, where *S_d_* is a sectional area of the dust particles. 

For example, in the case of a beam generated by an illuminating laser with transverse dimensions of 5 × 70 mm and a maximum power of 100 mW, this pressure is ≈10^−9^ Pa, while the corresponding force acting on a dust particle measuring 10 μm in size equals 0.79 × 10^−19^ N. For copper-coated particles with an illuminating laser power 36 mW, this force is almost three times lower. 

According to Figure 2, the threshold pressure acting on the particle at which a flow occurs is created by a cylindrical beam with a transverse size of 4 mm and power of ~75 mW. This pressure is 10^−7^ Pa and the corresponding force is 0.62 × 10^−17^ N.

Comparison of these values shows that the light pressure of the illuminating laser is two orders of magnitude weaker than the action of a pushing laser, at which the flow of uncoated particles occurs. Thus, the action of the illuminating laser cannot lead to the flow of a colloidal plasma medium.

**Figure 5:**
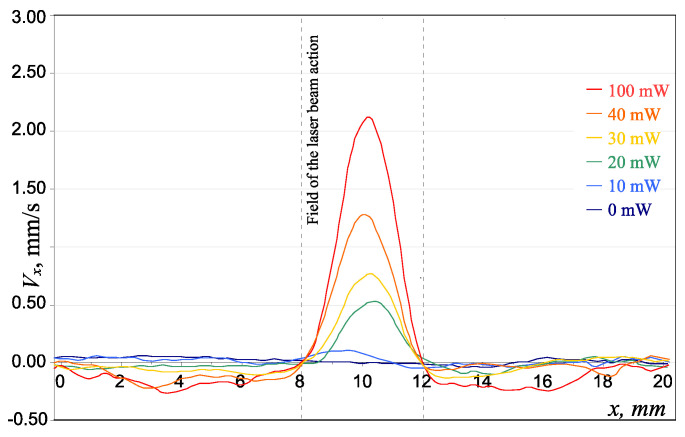
The velocity distribution profile for copper-coated MF particles in a monolayer dust structure under the action of a pushing laser with power values of W = 0, 10, 20, 30, 40, and 100 mW.

Now let us compare the force of the light pressure of the pushing laser and the photophoretic force created by it. In order to estimate the power of the light pressure in the case of coated particles, we set the coefficient *θ* to 0 in Relation (3), assuming complete absorption of the light flux. For coated particles, the maximum laser power used in our experiments was 100 mW, with a beam diameter of 4 mm. In this case, F_γ_ ≈ 0.83 × 10^−17^ N. This means that the force reached the values at which a laminar flow occurred for the uncoated particles. For the coated particles, as noted above, no visible evidence of flow of the dust component was observed (see Figure 4). At the same time, detailed image processing made it possible to detect an increase in the average particle velocity in the direction of a pushing laser beam (see Figure 5).

To estimate the photophoretic force, we used Relation (2). Assuming the thermal conductivity of the particles to be *æ* ≈ 400 W/(m·K), with all other conditions being equal, we find F_ph_ ≈ 0.92 × 10^−19^ N. This means that in the considered case, the maximum value of the photophoretic force is two orders of magnitude lower than the threshold, meaning this force should not cause a laminar flow in the dust structure. However, even with a relatively small power of the pushing laser (~20 mW), the kinetic heating of the dust structure in the area of the action of the pushing laser is visually observed, which noticeably increases with the growth of the beam power.

It should be noted that the photophoretic force at an illuminating laser power of W = 36 mW is only 0.3 × 10^−21^ N, which is two orders of magnitude less than the photophoretic force from a pushing laser of equivalent power. Nonetheless, even under the influence of such an insignificant photophoretic force, kinetic heating of the dust component occurs (see Figure 4).

### 2.4. Discussion

Taking all of the aforementioned information into consideration, we revealed completely different behavior for coated and uncoated particles in colloidal plasma under the action of laser radiation. For the uncoated MF particles, the light pressure effect occurred, leading to the appearance of a laminar flow. For the coated particles, we distinguished two action forces–light pressure and photophoretic force. Moreover, the actions behind these forces are significantly different. Light pressure causes some movement in the direction of the light beam, while the photophoretic force leads to heating and an increase of the kinetic energy, leading to chaotic movements of the dust component. However, the heating mechanism behind this phenomenon is still undetermined. Apparently, we must assume that there is a change of the direction of the particle movement when the laser radiation acts on the coated particles. This means that under the action of radiation, the particles deviate from the straight direction determined by the laser beam. Since the heating of the particles probably happens due to the photophoretic force (the force of light pressure acts strictly along the direction of the light beam and does not lead to the heating of the dust component of the plasma), we should note that this force is firstly estimated to be insufficient to form a laminar flow, and secondly the direction of its action does not coincide with the direction of the laser beam. A dusty particle in a gas discharge collides with electrons and ions, as well as with neutral particles from the buffer gas. During collisions, neutral atoms accumulate on the heated particle’s surface. Then, they leave the dust grain with greater speed, thus giving it an additional impulse. 

In addition, when evaluating the magnitude of the photophoretic force, we assumed that the coating was homogeneous and that there were no uncoated areas. However, if there are uncovered spots on the surfaces of the particles (see Figure 1), the temperature distribution can change dramatically, which will cause a significant increase in the photophoretic force. Therefore, the considered stochastic process, together with the inhomogeneity of the metal shell, can lead to asymmetry of the heating of the particles, resulting in chaotic deviation of the photophoretic force from the direction of the laser beam for different particles.

## 3. Experimental Setup

**Figure 6:**
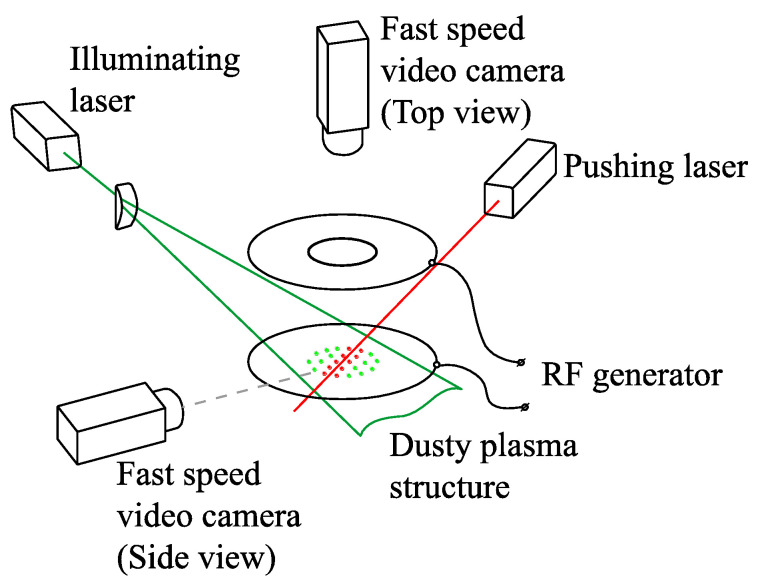
A scheme of the experimental setup for studying the colloidal plasma in a RF discharge under laser irradiation.

A scheme of the experimental setup for the studies of colloidal plasma in a RF discharge is presented in Figure 6. The main element is a vacuum chamber with optical windows, inside which two flat round electrodes are horizontally located at a distance of 5 cm from each other. There is a circular coaxial hole with a diameter of 6 cm in the upper electrode, above which the dust particle injector is placed, so that particles can fall through the hole to the discharge area. During the experiment, after pumping the chamber, it was filled with buffer gas up to a pressure of 3–7 Pa. In our experiments, we used argon as a plasma-forming gas. Generated plasma is highly non-isothermal. While the temperature of electrons is about 1–2 eV, the temperature of ions and atoms of a neutral gas is close to room temperature, i.e., ~300 K. As for the concentrations of electrons and ions, *n_e_* ≈ n_i_ ~ 10^8^–10^9^ cm^−3^.

From a high-frequency generator through an impedance-matching device, a voltage of 300 V with a frequency of 13.56 MHz was applied to the electrodes, resulting in ignition of glow discharge between them. To form a potential trap and prevent scattering of dust particles, a metal ring with a diameter of 51 mm was installed in the center of the lower electrode. The particles injected into the discharge acquired a negative charge and started levitating in the near-electrode layer, forming a monolayer dust structure.

To study experimentally the effect of laser irradiation on dust structures, we used the following configuration of the optical scheme (see Figure 6). For illumination of particles in the dust structure, we used a flat beam of an argon laser with a wavelength of 514 nm. A solid-state laser with a wavelength of 532 nm formed a beam with a diameter of 4 mm, which perturbed particles in the middle part of the structure so that the dust flow appeared. We chose lasers with minimal power fluctuations for better control and precise tuning of the laser exposure. Additionally, the laser radiation power and intensity profile in the beam, acting on the dust structure, were additionally monitored during the experiment.

The positions of the particles in the horizontal plane were recorded by means of a high-speed video camera (see Figure 6) located above the upper optical window of the experimental setup. To confirm that the monolayer was not degraded, we used a second video camera located in the horizontal plane. Obtained video data were processed using special software in order to determine the positions of individual dust particles in video frames. 

The power of the pushing laser was significantly higher than of the illuminating one. Inevitably, this could cause noticeable light flares on particles, which in turn might contribute to an error in the determination of the coordinates and velocities of analyzed particulates from the video recordings. To prevent this, lasers with different wavelengths and corresponding light filters in front of video cameras were used, which allowed us to either attenuate or eliminate the radiation of the pushing laser.

## 4. Conclusions

We present the results of an experimental study of the formation of a flow in a quasi-two-dimensional dusty structure in colloidal plasma. We observed different behaviors in the structure, depending on whether particles were coated or not. For “passive” uncoated particles that could not absorb laser radiation, a laminar flow channel formed in the exposed area. Moreover, this process had a laser power threshold, above which particle flow appeared. For particles with an absorbing copper-coated surface, an isotropic increase of kinetic energy was observed with the laser radiation power growth due to the presence of photophoretic force. However, for this case a flow channel was not visually detected. Nevertheless, an analysis of the velocity distribution profile indicated a non-isotropic increase of particle velocities along the direction of the laser beam action.
